# AMFormulaS: an intelligent retrieval system for traditional Chinese medicine formulas

**DOI:** 10.1186/s12911-021-01419-8

**Published:** 2021-07-30

**Authors:** Yidi Cui, Bo Gao, Lihong Liu, Jing Liu, Yan Zhu

**Affiliations:** 1 Zhejiang Academy of Traditional Chinese Medicine, Hangzhou, 310007 Zhejiang China; 2 Tongde Hospital of Zhejiang Province, Hangzhou, 310007 Zhejiang China; 3 Institute of Information on Traditional Chinese Medicine, China Academy of Chinese Medical Sciences, Beijing, 100700 China

**Keywords:** Traditional chinese medicine, Formula, Database, Retrieval system

## Abstract

**Background:**

Formula is an important means of traditional Chinese medicine (TCM) to treat diseases and has great research significance. There are many formula databases, but accessing rich information efficiently is difficult due to the small-scale data and lack of intelligent search engine.

**Methods:**

We selected 38,000 formulas from a semi-structured database, and then segmented text, extracted information, and standardized terms. After that, we constructed a structured formula database based on ontology and an intelligent retrieval engine by calculating the weight of decoction pieces of formulas.

**Results:**

The intelligent retrieval system named AMFormulaS (means Ancient and Modern Formula system) was constructed based on the structured database, ontology, and intelligent retrieval engine, so the retrieval and statistical analysis of formulas and decoction pieces were realized.

**Conclusions:**

AMFormulaS is a large-scale intelligent retrieval system which includes a mass of formula data, efficient information extraction system and search engine. AMFormulaS could provide users with efficient retrieval and comprehensive data support. At the same time, the statistical analysis of the system can enlighten scientific research ideas and support patent review as well as new drug research and development.

## Background

Traditional Chinese medicine (TCM) is a science that studies human life, health, and disease as well as a summary of the valuable experience of the Chinese nation in long-term survival and practice. Doctors of TCM treat patients based on syndrome differentiation by looking, listening, questioning, and feeling the pulse, then give TCM prescriptions to acquire the therapeutic effect. So, formula is an important therapeutic concept in TCM and always the research hotspot. There are many research directions of formulas, for example, the research about theory of formulas compatibility [1–4], the research about dosimetry of formulas[5, 6]; and the research about formula and disease [7–9].

However, knowledge of formulas is mainly recorded in diverse TCM books which results in the difficulties of retrieval and acquirement. Consequently, integrating formula information as well as constructing database can greatly improve the efficiency of retrieval, bring convenience of knowledge acquisition and utilization for researchers and clinical doctors. Nowadays, there are some formula databases, Guo et al. constructed a formula knowledge graph which presented the knowledge by the way of node-relation-node, and knowledge of the graph included traditional Chinese medicine, dosage, traditional Chinese Medicine processing, efficacy, and so on [10], Min He et al.constructed a traditional Chinese medicine database in the form of node and property, knowledge of the database included Chinese medicines, original plants, bioactive components, and the function of search and display were provided [11]. Both of the two databases store and display formula information based on the node-edge-node. However, the form of node-edge-node could only show the most important information by some terms rather than complete sentences, which might consult information loss; Shen et al.structured the listed proprietary Chinese medicine data and built the Chinese patent medicine database which integrated patent medicine information, but the patent medicine information was not sufficient for academic and clinical research [12; Ruichao Xue et al. constructed a traditional Chinese medicine integrative database to integrate the traditional Chinese medicine and western medicine which included some TCM knowledge, like formula, TCM drugs, and herbal ingredients. This database mainly focused on the herb molecular mechanism analysis, and didn't meet the needs of other formula research [13].

The above databases have some basic functions, like information retrieval and knowledge display. There are some limitations that need improvement, such as being small-scale and inaccessible to the original information. Meanwhile, with the development of computer technology, users tend to choose rich, strong correlation retrieval results in practical operation. To improve the efficiency of retrieval and utilization as well as realize the knowledge mining and discovery of formulas, we proposed an intelligent retrieval system, named AMFormulaS (means Ancient and Modern Formula system, 古今方药系统 in Chinese), which was based on a database containing a large number of formula and the relevant information of formula, like name of formula, composition, dosage of Chinese medicine and so on. The system also can efficiently extract formula information from the text data to extend the size of the database. In the meantime, we also proposed a method of weight calculation on formula drugs to improve the retrieval efficiency which compose the core part of the intelligent search engine.

## Methods

**Fig. 1:**
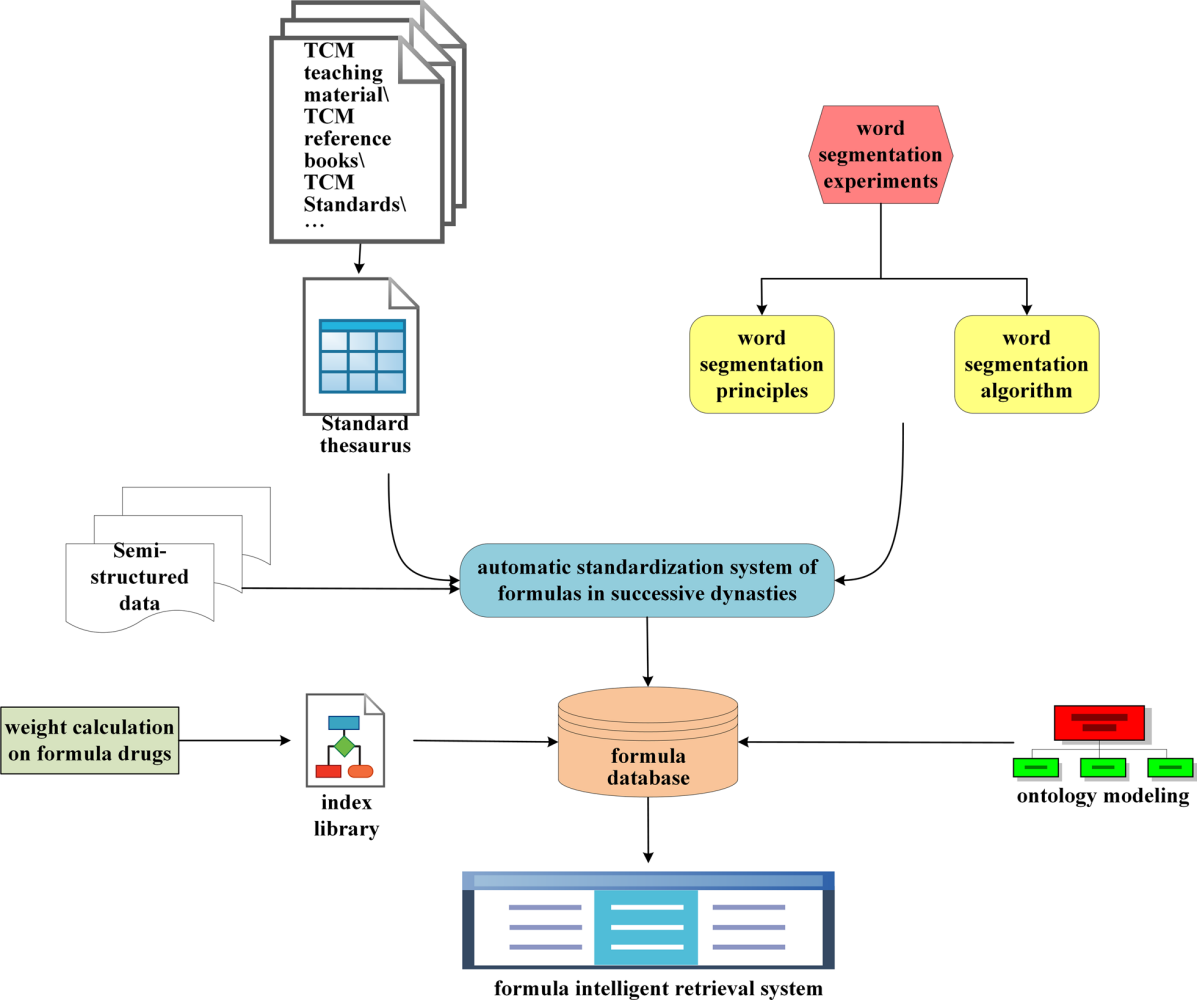
The pipeline of the construction about AMFormulaS

In the study, we firstly constructed an automatic standardization system that embedded the word segmentation packages and term dictionary. The semi-structured data was processed into structured and standardized formula records. A structured formula database was designed by incorporating the ontology modeling method. Meanwhile, we designed and implemented an algorithm of weight calculation on formula to improve the retrieval efficiency. Lastly, the formula intelligent retrieval system AMFormulaS was realized (See the pipeline in Fig. 1).

### Data sources

In this study, the data of AMFormulaS was selected and collected from the formula database maintained by the Institute of Information of Chinese medicine, Chinese academy of traditional Chinese medicine. It is a semi-structured database including 85,989 ancient and modern Chinese medicine formulas from more than 710 ancient books and modern literature. Considering the difference between ancient and modern medication habits, we took the modern medication habits as the standard reference and selected 38,000 formulas whose medicinal source of the component could be found or ascertained at present.

### Data processing

#### Word segmentation

Formula database involves a large amount of data, so it is necessary to use computer technology to improve the efficiency of data processing. Considering the huge workload and our existing researches, we decided to adopt the information extract solution of word segmentation algorithm integrated with large-scale terminology.

The ancient Chinese medicine text is distinctive in grammar and expression as well as professional terms. Therefore, the standard of word segmentation needed to be investigated and determined initially. In one of our previous studies [14], we constructed a corpus for training the algorithm of word segmentation. According to the classification method of *traditional Chinese medicine philology* [15], we firstly selected 30 TCM ancient books of Qing Dynasty involving 10 categories: Materia medica, formulas, febrile diseases, internal medicine, surgery, gynecology, pediatrics, facial features, acupuncture and massage, medical cases respectively, and manually selected 150 pieces of rough corpus which contained 1705 sentences and 88,889 words from these books to train the model. Then the selected corpus was tagged manually by referencing TCM teaching material for higher education students [16], TCM reference books [17], and TCM related standard [18, 19] as terminology sources. After that, we preliminarily summarized the standard of word segmentation in TCM ancient books, that was taking the existing facts and semantic changes as the primary principle and considering the principles of part-of-speech grammar and semantic type in the meantime. There were 17 semantic types of text which were segmented based on the principle including physiology, symptom, syndrome, pathological factors, pathological products, efficacy, method of treatment, channel meridian and acupuncture points, four diagnostic methods, traditional Chinese drug, prescription, nature and flavor, toxicity, processing, contraindications, decoction method, and proprietary words in Chinese medicine.

After manual labeling the training set of word segmentation, we trained a model based on the algorithm of capsule network [20]. Compared with other algorithms, the algorithm of capsule network showed a good performance for word segmentation in ancient traditional Chinese medicine literature, so the capsule network model was used for word segmentation.

#### Information extraction and standardization

**Fig. 2:**
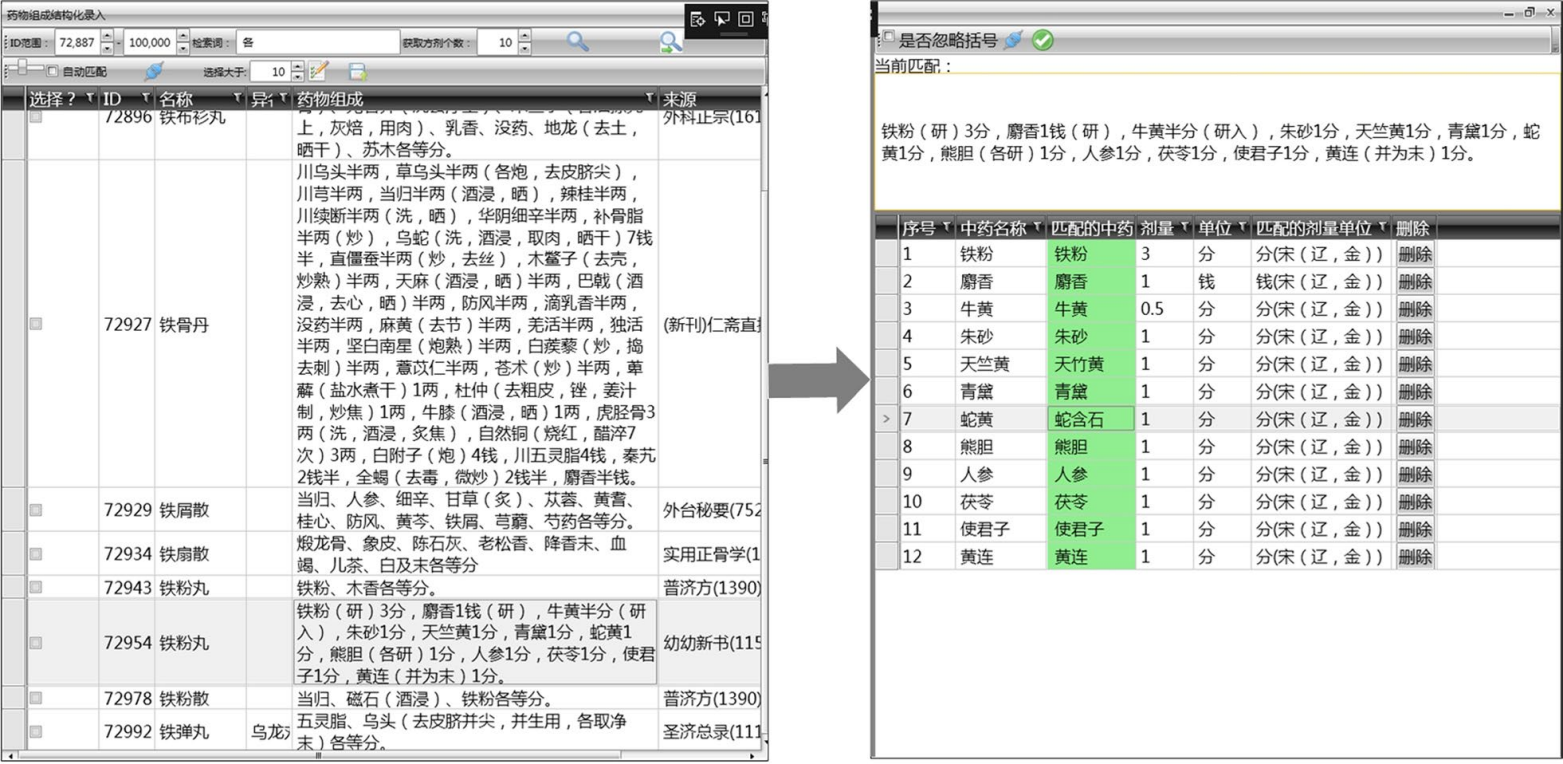
The information extraction and standardization of Tiefen pellet

Due to the heterogeneity in the data structures and lack of standards for formula information, we built a system named automatic standardization system of formulas in successive dynasties to extract and standardize the information of formulas under the guidance of the above-mentioned word segmentation standard and algorithm. The system firstly realized the identification, extraction, and standardization of formula, then the processed data was submitted to the formula database after manual verification. The extracted and standardized content includes name, composition, source, formation year of formulas, the dose of decoction pieces, the processing method of natural crude Chinese medicine, etc.[21]. For example, the formula of Tiefen pellet (铁粉丸 in Chinese) comes from the book *You you xin shu *(《幼幼新书》in Chinese) written in the Song Dynasty. The system could transform the text into structured data. Firstly, the system recognized the information of the formula in the form of text, like name of the formula, traditional Chinese medicine, dosage, and then extracted and standardized this information. For instance, one of the components is “Shehuang(蛇黄 in Chinese)” in the original records, the system identified it and normalized to “Shehanshi(蛇含石 in Chinese)”, the dosage and the measuring unit also could be normalized (as shown in Fig. 2).

#### Design of formula database

**Fig. 3:**
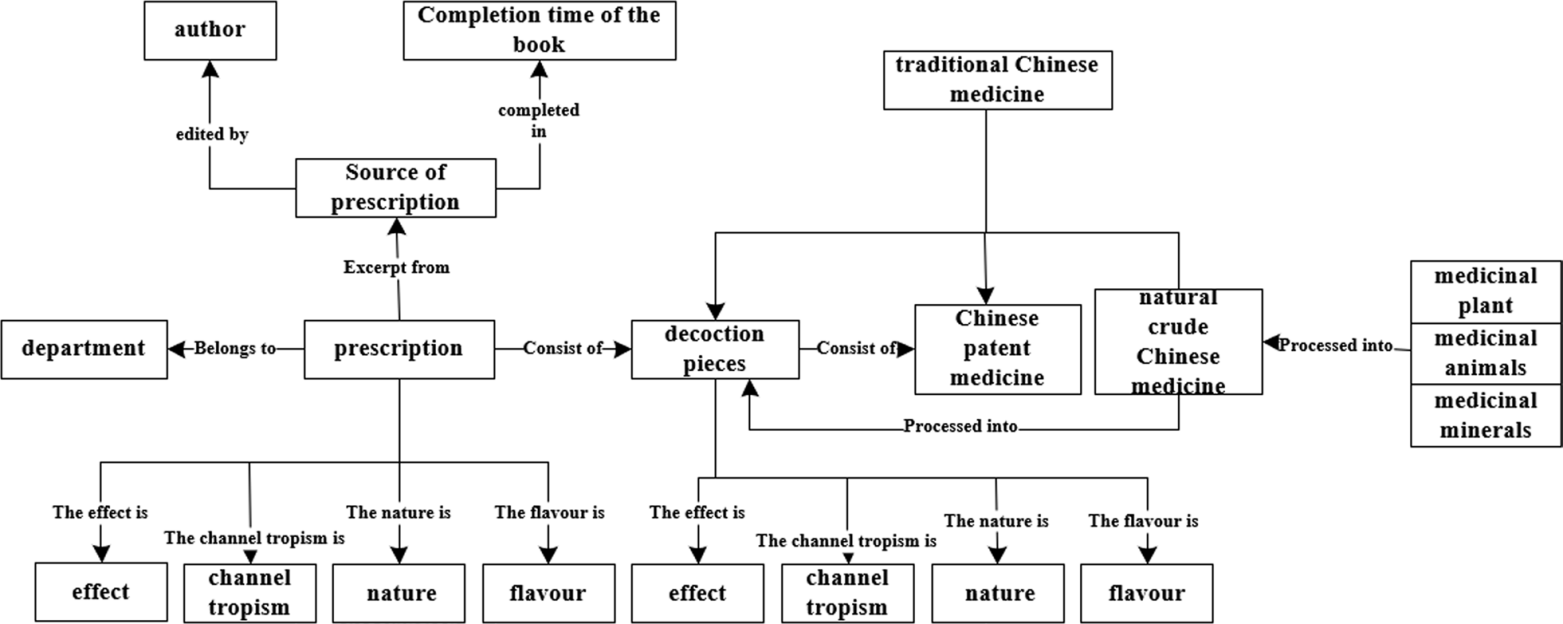
Conceptual data model of formula database

There is a variety of information about formulas, therefore, all contents of the formula database should be completed and well-organized including formula name, source of formula, formation date, author, and composition of formula, etc. Employed our formal research [22, 23], on the ontology-based modeling, the concept, relation, and property were analyzed and determined, then schema of formulas database was designed based on the conceptual modeling method of ontology and the authoritative references of TCM, such as Pharmacopoeia of the *People's Republic of China (part 1)* [24], *Coding Rules and Codes of Traditional Chinese Medicine* [25], *Chinese materia medica* [26], *Dictionary of traditional Chinese medicine* [27], etc. The entities of the ontology model contain the information of formula (name, source, author, the subordinate departments, effect, and nature, flavor and channel tropism), information about Chinese medicine (medicinal name, medicinal sources, effect, Chinese patent medicine, decoction pieces, and effect and nature, flavor and channel tropism), the core concept graph of formula database is shown in Fig. 3.

#### Implementation of the intelligent retrieval system

There exist some formula databases or retrieval systems that integrate some formula information, most of which only support full-text retrieval or retrieval by keyword. Yet, formulas are composed based on the TCM theory named Monarch, Minister, Assistant and Guide (君臣佐使 in Chinese). In the context of drug retrieval, users prefer to get the results which the search term of composition herb plays an important role. For example, by inputting ‘processed licorice (炙甘草 in Chinese)’, the users usually expect the result including formulas in which processed licorice play the Monarch role. Hence, a method of weight calculation on formula drugs was proposed in this research which made results be sorted by the importance of decoction pieces in formula or formation time of formula [28]. In this research, three factors were included to calculate the weight of the drug composition in the formula:

1. Whether the decoction piece is part of the name of the prescription which is processed by string matching;
2. The relative dose of traditional Chinese medicine. The relative dose was calculated as:
  1 $$f(o|S) = \frac{1}{n}\sum\limits_{i = 1}^{n} {G_{h} (t,t_{i} )} = \frac{1}{{n\sqrt {2\pi } }}\sum\limits_{i = 1}^{n} {e^{{ - \frac{{d(t,t_{i} )^{2} }}{{2h^{2} }}}} }$$
  where *d(t, t*_*i*_*)*^*2*^ represents the dose-distance between tuples *t*, *t*_*i*_,$G_{h} (t,t_{i} ) = \frac{1}{{\sqrt {2\pi } }}e^{{ - \frac{{d(t,t_{i} )^{2} }}{{2h^{2} }}}}$ is the Gaussian kernel function, *n* represents the number of different doses (or dose intervals) in T.
3. Whether decoction pieces are commonly used. The weight was calculated as:
  2 $$w(t) = \log (n/f(t))$$
  where *w(t)* is the weight of decoction piece *t*, *n* is the number of all different decoction pieces in set S, *f(t)* is the number of formulations containing the specified decoction pieces *t.*
4. Multiple linear regression was used to calculate the optimal parameters:
  3 $$y = w_{0} + x_{1} w_{1} + \cdots + x_{i} w_{i} + \cdots + x_{n} w_{n}$$
  where *x*_*1*_: Whether the drug is commonly used, that is, the occurrence frequency of the drug; *x*_*2*_: Whether the drug appears in the drug name; *x*_*3*_: The ratio of the dose used to the general dose of the drug.

Based on a training data set of 400 records (part of the experimental results shown in Table1), the obtained training parameters were:

$$w_{1} = 52.8231, w_{2} = 0.8773,w_{3} = 0.0470;w_{0} = 3.0705$$

**Table 1:** Part of the experimental results of parameter calculation

Formula ID	Formula	Decoction piece ID	Decoction piece	Annotated weight	X_1_	X_2_	X_3_
2	Daqinjia-o Powder	19,153	Gentiana macroph-ylla	4	0.00064414	1	0.066
6	Damangcao Pow-der	19,381	shikimic	4	0.00052605	1	0.0523
14	Datong Pills	17,578	cinnabar	4	0.00228797	0	0.0224
14	Datong Pills	19,988	clove	3	0.00022269	0	0.0266
14	Datong Pills	17,878	lead powder	3	0.00022269	0	0.038
15	Datong Pills	17,591	dendrob	2	0.00028504	0	0.098
15	Datong Pills	19,972	ginseng	4	0.00248455	0	0.0349
18	Datong Pills	17,546	tatarian aster	3	0.00487358	0	0.1042
19	Datong Pills	17,820	niter	3	0.00156434	0	0.0529

The standard deviation between the predicted result and the labeled result was 0.719, and the error was within the acceptable range. Then the algorithm was applied to the intelligent search engine system of formula.

Besides, other retrieval functionalities also were implemented, including:

1. Full-text retrieval: link to the index base according to the search terms and realize the global retrieval by the keywords.
2. Precise retrieval: by different semantic types of search terms to achieve precise retrieval including:
  - by decoction pieces
  - by Chinese crude drug
  - by the creation time of formula
  - by the department of formula
  - by the classification of the formula efficacy
  - by the nature, flavor, and meridian tropism of the formula
  - combination of full text and precise retrieval: by keywords and semantic entries

## Results

**Fig. 4:**
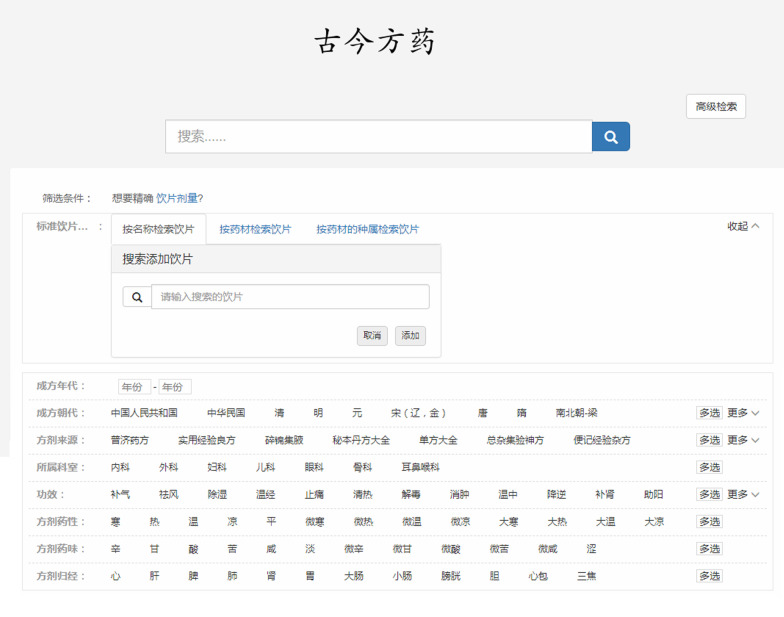
The home page of AMFormulaS

AMFormulaS was developed based on B/S architecture, Java language, and MySql5.7, composed by modules of information retrieval of formulas, decoction pieces and Chinese crude drug, statistical analysis, and visualization (the home page of the retrieval system is shown in Fig. 4). On the search results page, users can not only browse the specific information of formulas, the related information of decoction pieces and decoction pieces combination, but also the global statistical information of decoction pieces and formulas in the whole database. Users can search relevant information according to their needs and select the appropriate presentation pages, such as formula retrieve, decoction pieces retrieve, decoction pieces combination retrieve, and Dashboard.

### Formula retrieval

**Fig. 5:**
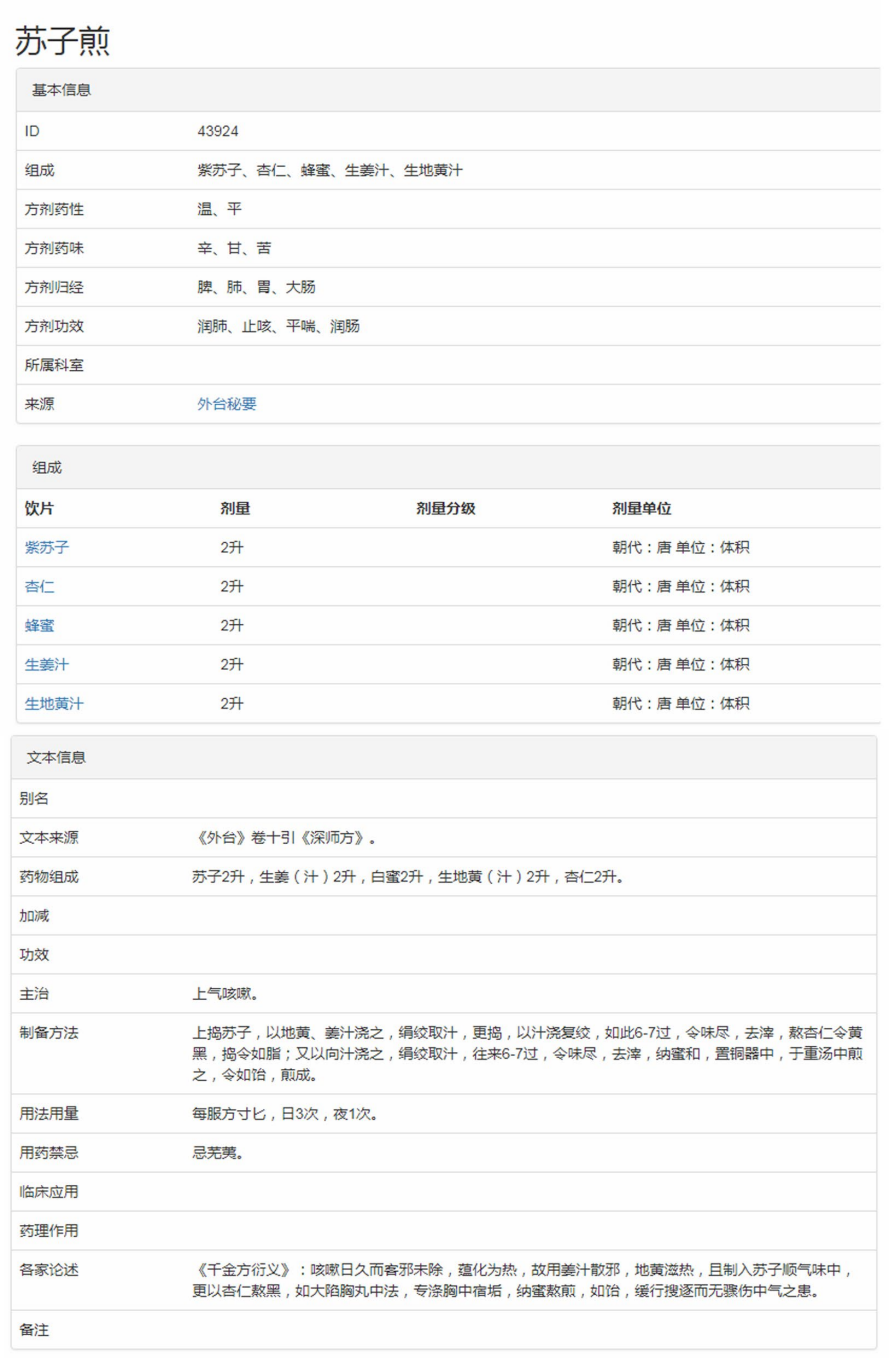
The retrieval results of Suzi Decoction

**Fig. 6:**
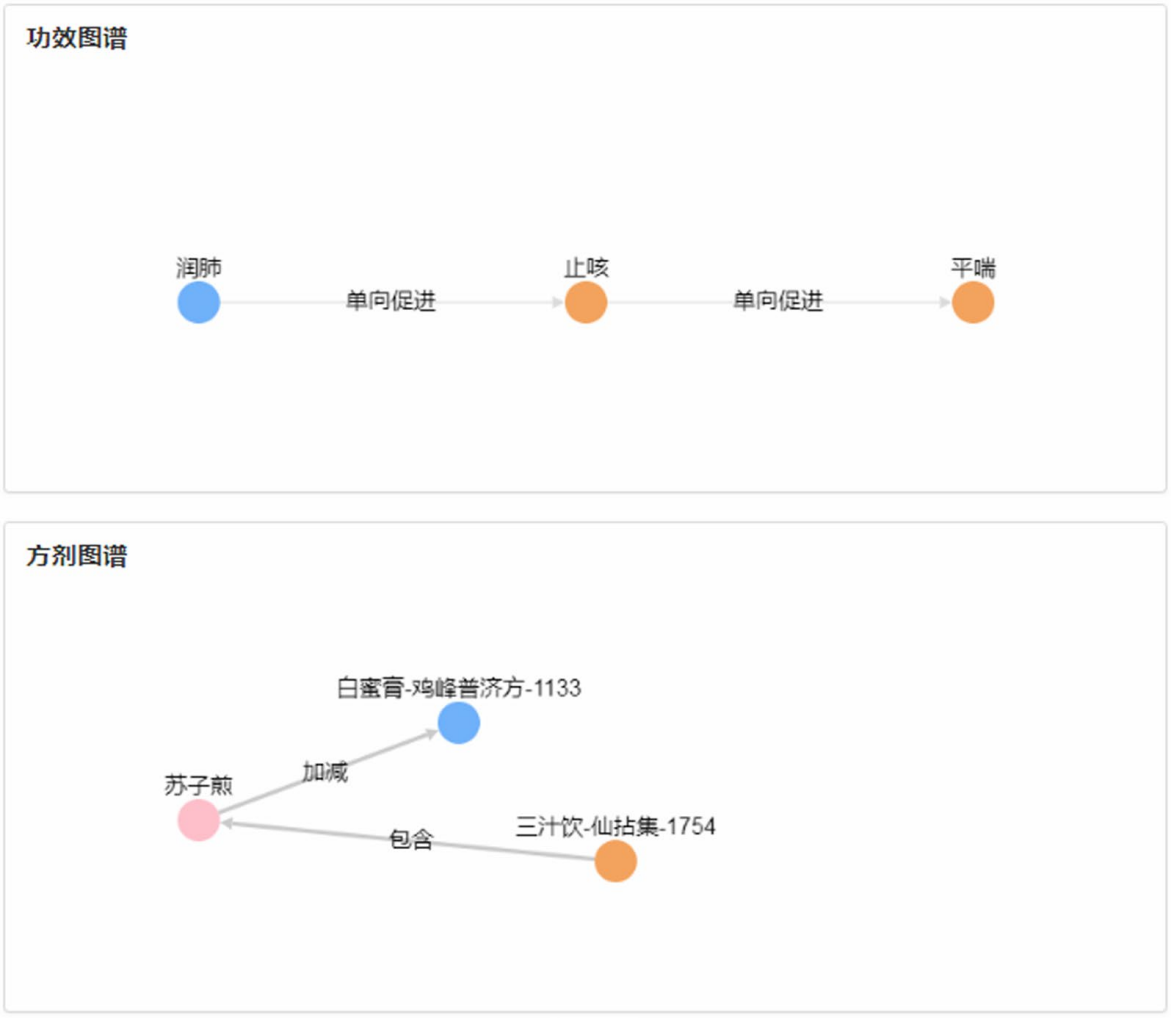
The efficacy relation and the graph of addition or subtraction of drugs on Suzi Decoction

By the entered name of the formula, the information of formulas will be displayed on the page including an ID of the formula, composition, efficacy, nature, flavor and channel tropism, department, source, formation time as well as the original text information of the formula. A formula is made up of decoction pieces, the addition or subtraction of drugs lead to continuous changes of formula, like name, efficacy. Take Suzi Decoction for example, the retrieval results are shown in Fig. 5. The efficacy relation and graph about the addition or subtraction of composition drugs are shown in Fig. 6.

### Decoction pieces retrieval

**Fig. 7:**
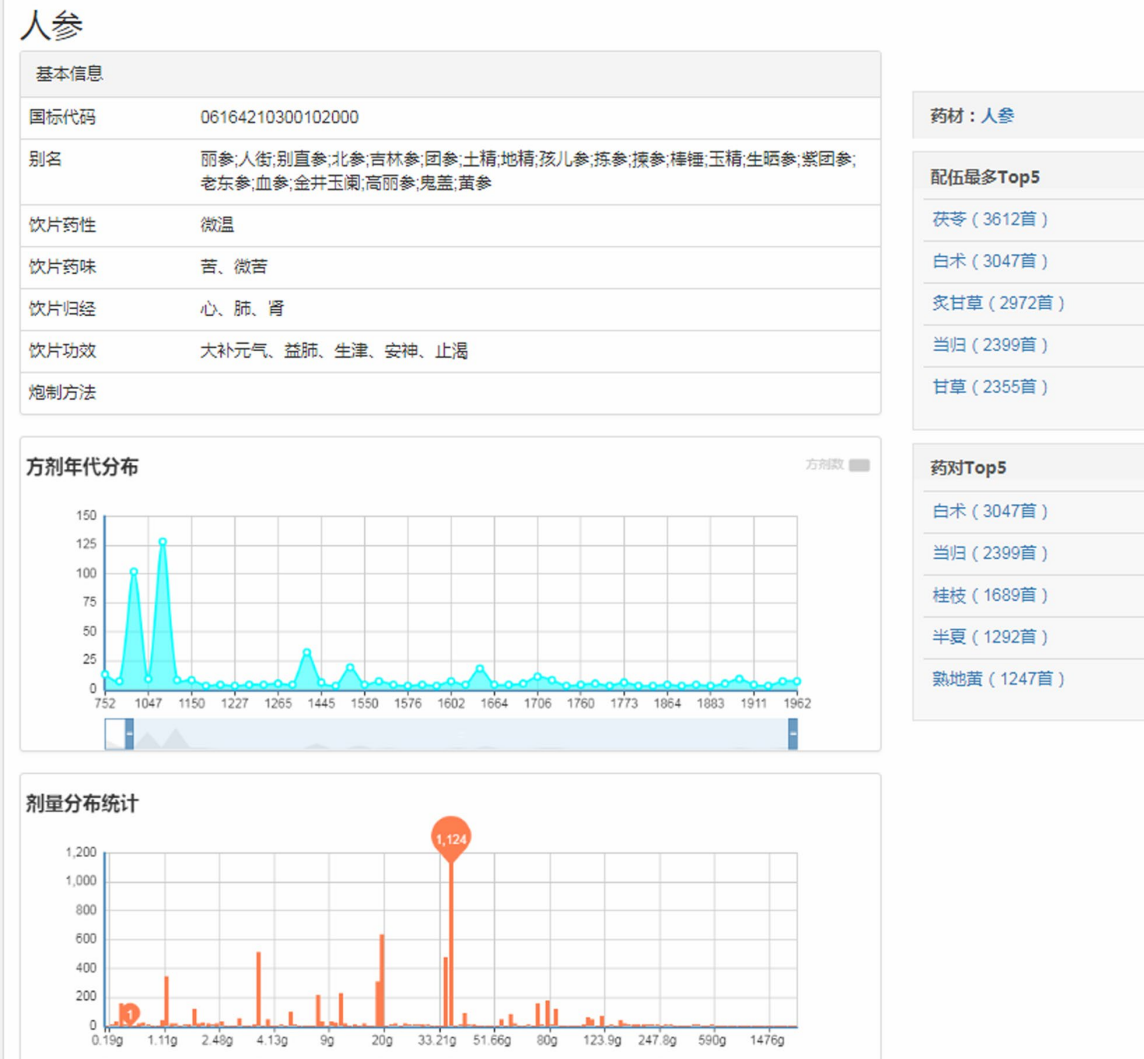
The decoction pieces retrieval results of ginseng

By the inputted name of a decoction piece, the system will search and return the basic information, time distribution of formulas containing the decoction piece, and the use frequency about the diverse dosage of the decoction piece. Take ginseng for example, the retrieval results are shown in Fig. 7.

### Retrieval of decoction pieces combination

**Fig. 8:**
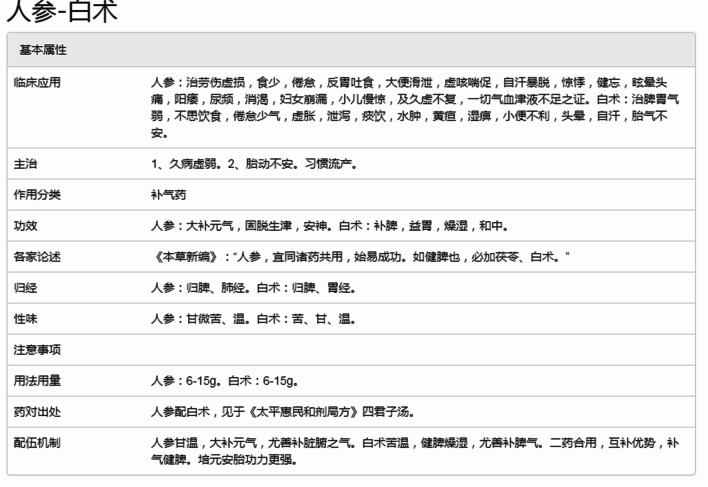
The basic information about the combination of ginseng and largehead atractylodes rhizome

The system supports the query of the combination of decoction pieces. By the entered name of decoction pieces, the system can display the basic information of the decoction pieces combination, like clinical application, indications, action classification, efficacy, compatibility of the combination, etc. (as shown in Fig. 8).

**Fig. 9:**
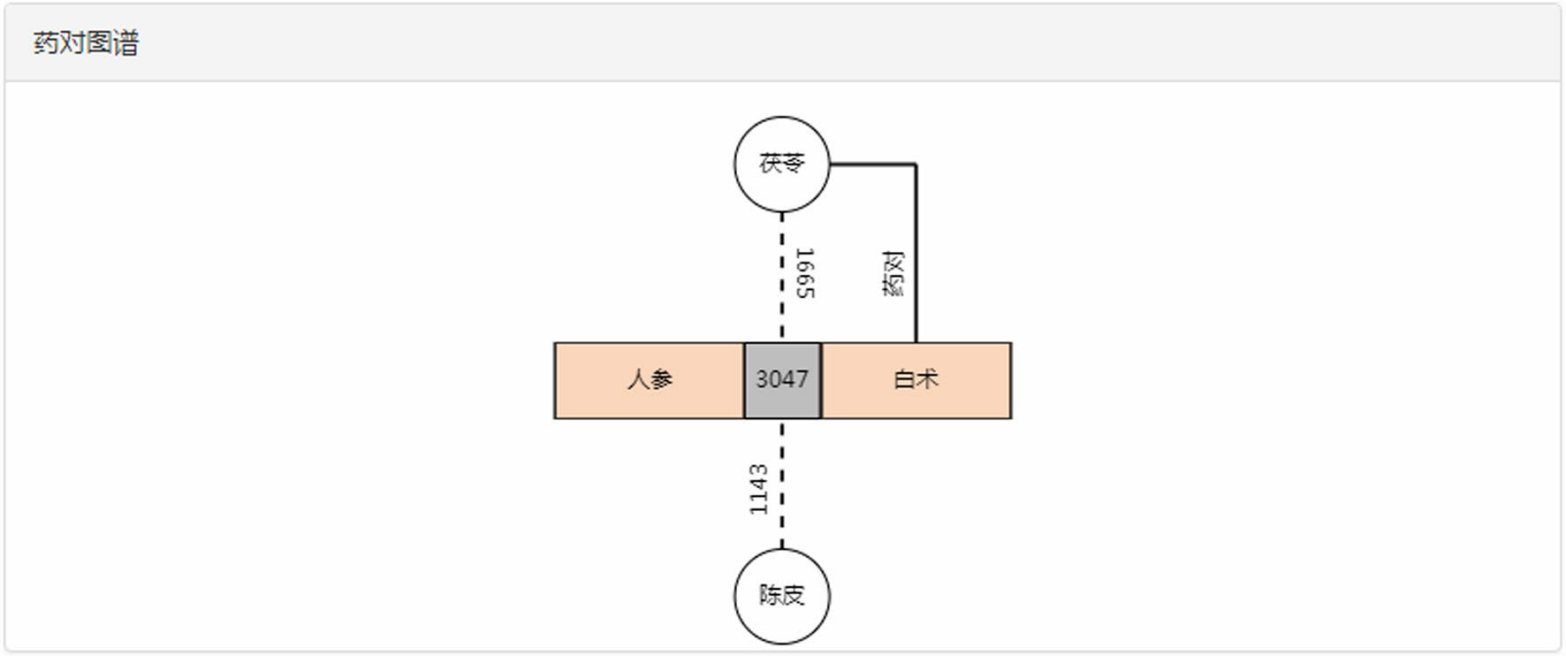
The compatibility and frequency of ginseng and largehead atractylodes rhizome

Figure 9 shows rich information about relations between the formulas and the combination of ginseng (人参 in Chinese) and largehead atractylodes rhizome (白术 in Chinese). Such as: (1) ginseng and largehead atractylodes rhizome both appear in 3,047 formulas; (2) ginseng, largehead atractylodes rhizome and Indian bread (茯苓 in Chinese) appeared in 1,665 formulas, and (3) ginseng, largehead atractylodes rhizome and dried tangerine peel (陈皮 in Chinese) appeared in 1,143 formulas.

**Fig. 10:**
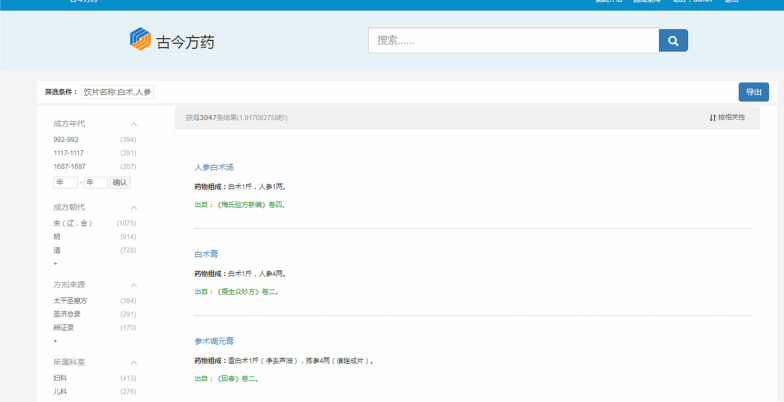
The retrieval results about the combination of ginseng and largehead atractylodes rhizome

**Fig. 11:**
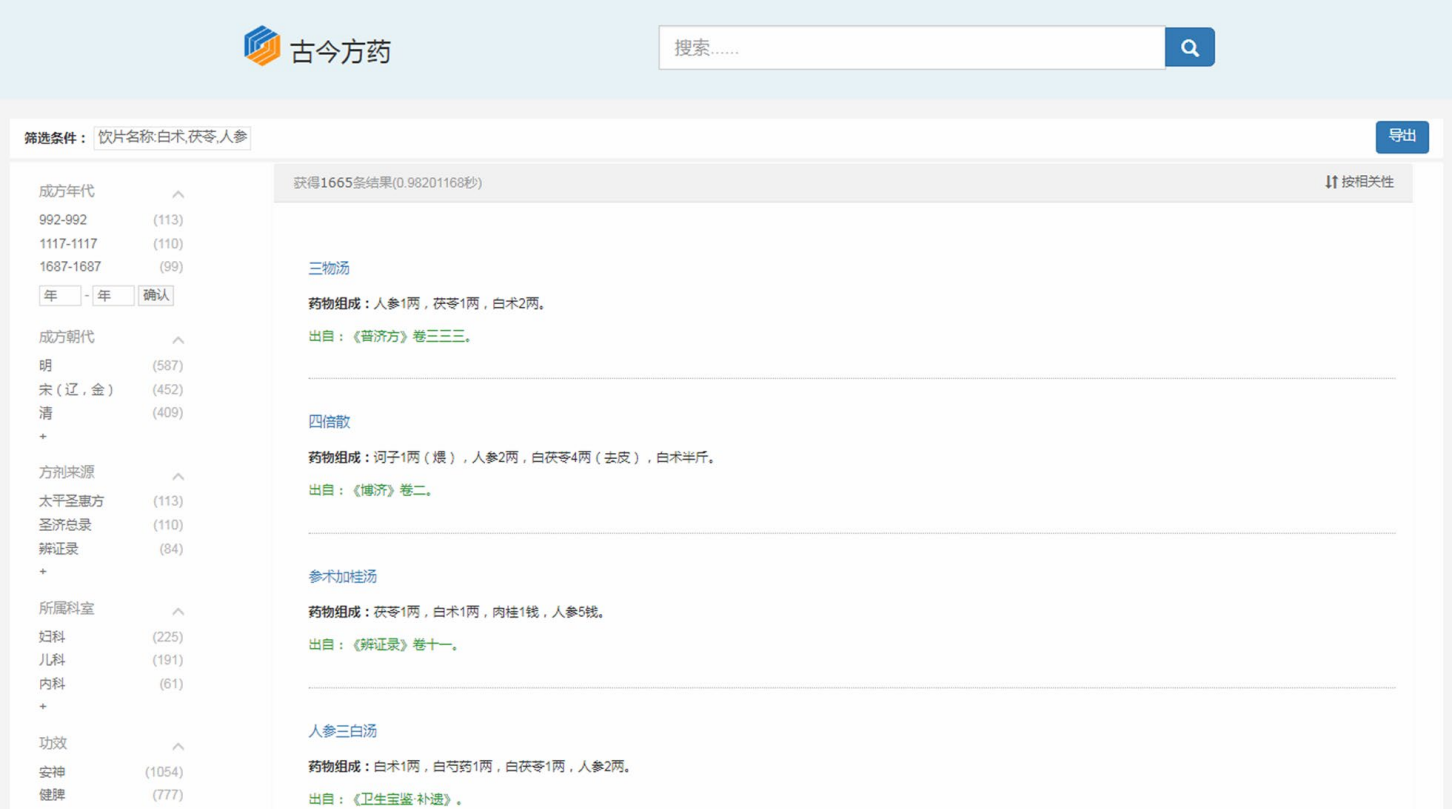
The retrieval results about the combination of ginseng, largehead atractylodes rhizome

Besides, the retrieved formulas containing these decoction pieces can be looked up and sorted according to the importance of the combinations of these decoction pieces in formulas. For instance, by inputting “ginseng” and “largehead atractylodes rhizome”, there are 3,047 formulas that can be retrieved. After intelligent sorting, the first formula shown to users is "Renshenbaizhu soup" (as shown in Fig. 10). In the same light, when searching “ginseng”, “largehead atractylodes rhizome" and “Indian bread”, the first formula is "Sanwu soup" (as shown in Fig. 11).

### Dashboard

**Fig. 12:**
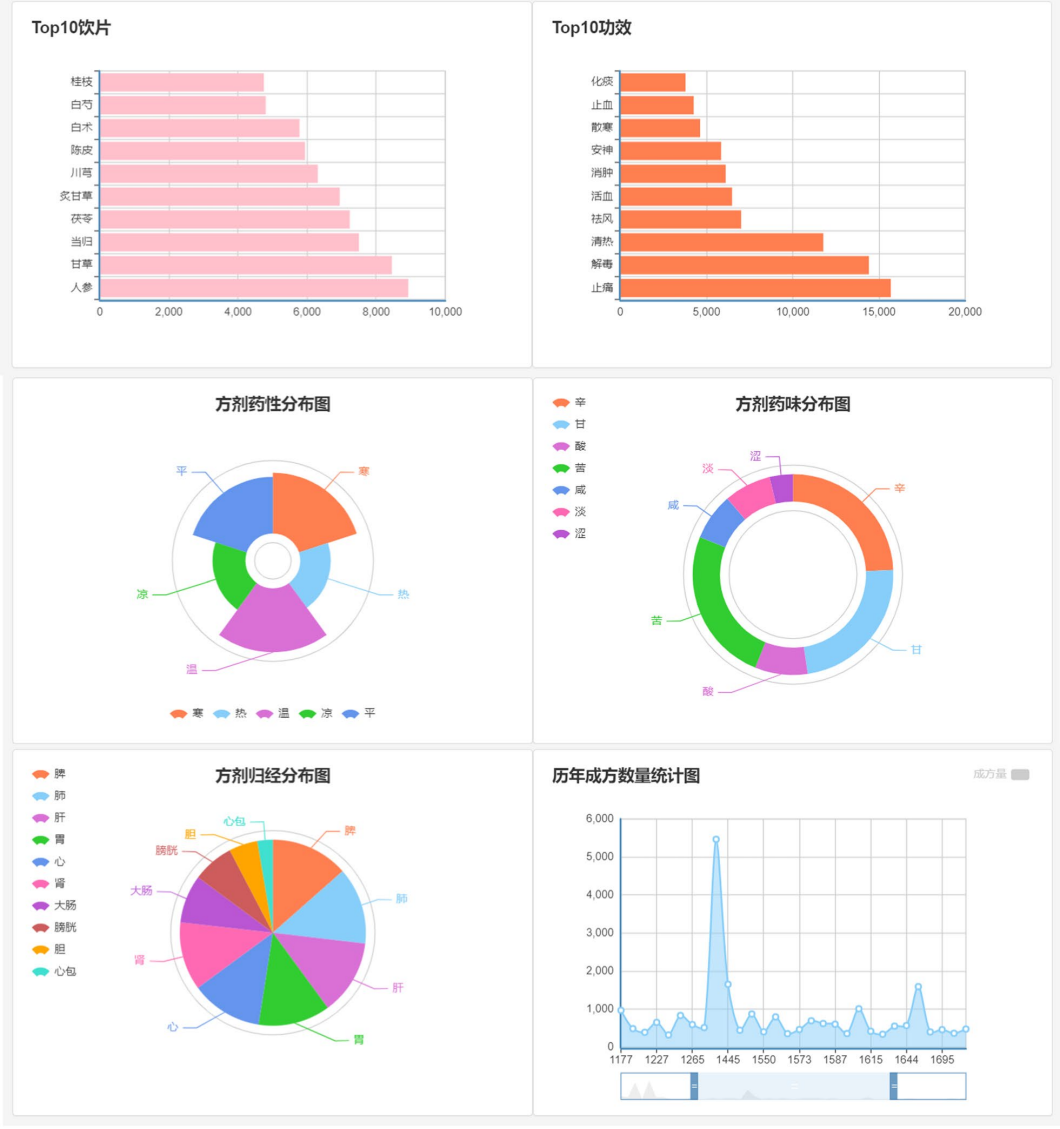
Dashboard of AMFormulaS

The dashboard shows the statistics about all the Chinese medicine decoction pieces and formulas in the database, such as the top 10 decoction pieces and efficacy of formulas appeared in this database, the statistics about nature, flavor and channel tropism of formula and the number of formulas formed in every dynasty (as shown in Fig. 12).

## Discussion

AMFormulas aims at sorting formula information and providing intelligent retrieval services for medical staff, researchers, and students, at the same time, providing data support for the generation of class formulas, screening of classic formulas, data mining of formulas, as well as new drug research and development. Based on integrating the formula data, the system makes a multi-dimensional statistical analysis of the formation time of formula, medication frequency, dosage of formulas and drugs in the past dynasties. The data and analytical results about the time, medication habits, dosage of traditional Chinese medicine to help enlighten many new research directions. Meanwhile, the system can provide comprehensive and accurate intelligent query services for patent application and protection.

Considering the knowledge of formulas involves a wide scope and enormous quantity, more formulas need be included in the future. The current version of AMFormulaS only aims at verification for system design and retrieval algorithm. As the scale of database and users growing, more tests and updates on performance will carried out to meet users’ needs of more accurate retrieval engine, high-quality data, and other services.

## Conclusions

In this study, a total of 38,000 formulas were structured and standardized through information extraction methods, then imported into the structured formula database. A novel intelligent formula retrieval system, AMFormulas, was built capable of multi-dimensional retrieval, and statistical analysis of formula information. The system collected, standardized, and integrated a large amount of formula information, including the original text of formulas. It not only realizes efficient retrieval and statistical analysis but also enables the users to access the original data source.

## Data Availability

The data that supporting the findings of this study are available from the corresponding author on request.

## Abbreviations

AMFormulaS: Ancient and Modern Formula System
TCM: Traditional Chinese medicine
